# Fourier Transform Infrared Microscopy Enables Guidance of Automated Mass Spectrometry Imaging to Predefined Tissue Morphologies

**DOI:** 10.1038/s41598-017-18477-6

**Published:** 2018-01-10

**Authors:** Jan-Hinrich Rabe, Denis A. Sammour, Sandra Schulz, Bogdan Munteanu, Martina Ott, Katharina Ochs, Peter Hohenberger, Alexander Marx, Michael Platten, Christiane A. Opitz, Daniel S. Ory, Carsten Hopf

**Affiliations:** 1 Center for Applied Research in Applied Biomedical Mass Spectrometry (ABIMAS), Mannheim University of Applied Sciences, Paul-Wittsack Str. 10, 68163 Mannheim, Germany; 2 Institute of Medical Technology, Heidelberg University and Mannheim University of Applied Sciences, Paul-Wittsack Str. 10, 68163 Mannheim, Germany; 3 German Cancer Consortium (DKTK) CCU Neuroimmunology and Brain Tumor Immunology, German Cancer Research Center (DKFZ), Heidelberg, Germany; 4 University Medical Center Mannheim of Heidelberg University, Mannheim, Germany; 5 Brain Cancer Metabolism Group, German Cancer Research Center (DKFZ), Heidelberg, Germany; 6 Department of Neurology and National Center of Tumor Diseases, University Hospital Heidelberg, Heidelberg, Germany; 7 Diabetic Cardiovascular Disease Center and Department of Medicine, Washington University School of Medicine, St. Louis, Missouri 63110 USA

## Abstract

Multimodal imaging combines complementary platforms for spatially resolved tissue analysis that are poised for application in life science and personalized medicine. Unlike established clinical *in vivo* multimodality imaging, automated workflows for in-depth multimodal molecular *ex vivo* tissue analysis that combine the speed and ease of spectroscopic imaging with molecular details provided by mass spectrometry imaging (MSI) are lagging behind. Here, we present an integrated approach that utilizes non-destructive Fourier transform infrared (FTIR) microscopy and matrix assisted laser desorption/ionization (MALDI) MSI for analysing single-slide tissue specimen. We show that FTIR microscopy can automatically guide high-resolution MSI data acquisition and interpretation without requiring prior histopathological tissue annotation, thus circumventing potential human-annotation-bias while achieving >90% reductions of data load and acquisition time. We apply FTIR imaging as an upstream modality to improve accuracy of tissue-morphology detection and to retrieve diagnostic molecular signatures in an automated, unbiased and spatially aware manner. We show the general applicability of multimodal FTIR-guided MALDI-MSI by demonstrating precise tumor localization in mouse brain bearing glioma xenografts and in human primary gastrointestinal stromal tumors. Finally, the presented multimodal tissue analysis method allows for morphology-sensitive lipid signature retrieval from brains of mice suffering from lipidosis caused by Niemann-Pick type C disease.

## Introduction

In recent years preclinical research and clinical practice have been substantially advanced by *in vivo* multimodality imaging approaches that combine multiple sensor types, for example diffusion-weighted magnetic resonance imaging (MRI) with computed tomography (CT) or positron emission tomography/CT^1–3^. Concomitantly, the popularity of multimodal diagnostic tissue analysis has been fueling the growth of unprecedented data volumes. In an analogous manner, initial multimodal *ex vivo* molecular tissue analysis methods have recently been presented, which emphasize the combination of MRI or photonic spectroscopy with mass spectrometry imaging^4–10^. To address the resulting computational challenges, most studies so far have focused on multimodal image registration, data fusion and subsequent analysis of the extracted hybrid sensory features^2,3,11,12^. However, multimodal data registration and image fusion typically lead to substantial increases in acquisition time and data load, which renders fusion of ultra-high dimensional data, such as Fourier transform ion cyclotron resonance (FTICR) MS images, with spectroscopic images impracticable. Moreover, final interpretation of the resulting fusion images is eventually left to a scientist’s or physician’s annotation that is typically based on only a small part of the acquired data. Consequently, another concept of multimodality is currently emerging, according to which one modality such as manual annotation of digitized H&E stained slices predefines advantageous features (such as spatial distribution patterns), which are then used to guide data acquisition and interpretation by another complementary modality^13–17^. This extension of the general multimodal imaging concept holds the potential to drastically decrease data volumes and, hence, computational cost. However, for successful translation to clinical application, current methods need automation to enable the use of commercially available high-content imaging instruments. Current concepts either rely on manually annotated histopathology^14^ of adjacent H&E stained tissue sections or utilize polarimetry imaging maps generated by a home-built instrument^13,15,16^ to manually define rectangular regions of interest (ROIs).

FTIR imaging uses commercially available instruments and has the ability to record general biomolecular characteristics of an unstained tissue (e.g. vibrational spectroscopic bands characteristic of lipids, proteins or nucleic acids) in a rapid, label-free and non-destructive manner^18,19^. The technique has gained recognition as a diagnostic tool revealing disease-related structural and compositional features in various cancer types and other diseases such as diabetes mellitus^20–25^. It is one of several techniques that are referred to as clinical spectroscopy^26^. Moreover, FTIR imaging has been effectively used in guiding laser capture microdissection (LCMD) in an automated setup to spatially resolve and extract malignant mesothelioma, which is characterized by a high degree of heterogeneity^17,23^. FTIR imaging may therefore be particularly well suited as the guiding upstream modality for combination with MALDI-MS imaging that is deployed as the complementary downstream modality. MALDI-MSI is widely used in biological and medical research areas, as it is able to monitor the distribution of thousands of biomolecules, i.e. chemically specific profiles of lipids and metabolites, to supplement conventional histopathology^27–29^. As high specificity is mandatory for MALDI-MSI, recent developments have focused on improved spatial resolution, resolving power and mass accuracy^30^. However, the resulting increased data load and acquisition time limit throughput of MALDI-MSI. Although various data mining approaches have been proposed to address the “big data” challenges of MALDI-MSI by means of supervised classification models^31,32^ and multimodality-concepts^12,14,15^, additional approaches to reduce data load are needed.

Here, we present an integrated workflow that combines FTIR microscopy imaging and complementary MALDI-MSI of a single tissue slice for automated, guided and spatially restricted MSI data acquisition and interpretation that does not require histology stains or other pre-processing steps. While FTIR imaging does not require any tissue processing and thus avoids possible MALDI-prototypical distortions introduced by solvent extraction and co-crystallization with a chemical matrix, it provides high-resolution structural and compositional information of a tissue without revealing individual biomolecular compounds. By contrast, high-resolution MALDI-MSI allows for chemically specific characterization of the underlying molecules based on mass-to-charge ratios (*m/z*). The workflow presented enables specification of tissue morphologies prior to MSI data acquisition by means of FTIR data segmentation with a superior accuracy when compared to MSI, thus allowing for an unbiased tissue-specific guidance without relying on prior pathological annotation. Moreover, the presented pipeline enables automated, spatially aware retrieval of molecular MSI signatures, as well as a drastic decrease in data load and acquisition time. We exemplify the general applicability of this integrated workflow by unsupervised tumor localization and marker extraction in CD1 nu/nu mice engrafted with U87 glioma tumor cells^33^ and in primary human gastrointestinal stroma tumors^34^. Furthermore, we show segment-specific lipid-disorders in Niemann-Pick C1 (NPC1) I1061T knock-in mice^35^ and introduce targeted MS imaging of small tissue sub-compartments by FTIR-guided high-resolution MALDI-FTICR-MSI, thus reducing data load and acquisition time by >97%. The obtained results emphasize the wide application potential of our workflow to guide molecular *ex vivo* in-depth tissue analysis.

## Results

A workflow for automated histopathology-independent FTIR-based tissue segmentation and targeted MS imaging from single-slice tissue specimen using commercially available instruments. In this study, we sought to develop a novel workflow that should employ commercially available instruments to acquire multimodal images of the same tissue section. Segments computed from one image should then be transferred to the mass spectrometer in an automated fashion to provide (i) guided ROI definition prior to MS image acquisition as well as (ii) unbiased exploration of (segment-specific) mass features after MS image acquisition.

To this end, we chose FTIR microscopy imaging as the guiding modality for its capability of recording distribution patterns of molecular vibrations in a non-destructive manner at a pixel size limit of 6.25 × 6.25 µm. For lack of interference of water bands in mid-infrared spectra, FTIR imaging can be carried out immediately after mounting of tissue on gold slides without any additional pre-processing steps. Moreover, FTIR images are devoid of distortions such as ion suppression or co-crystallization-borne inhomogeneity common to MALDI-MSI, and they represent molecular disease-related features, e.g. amide-bond vibrations or P-O-bond vibrations characteristic of proteins or nucleic acids, respectively.

**Figure 1:**
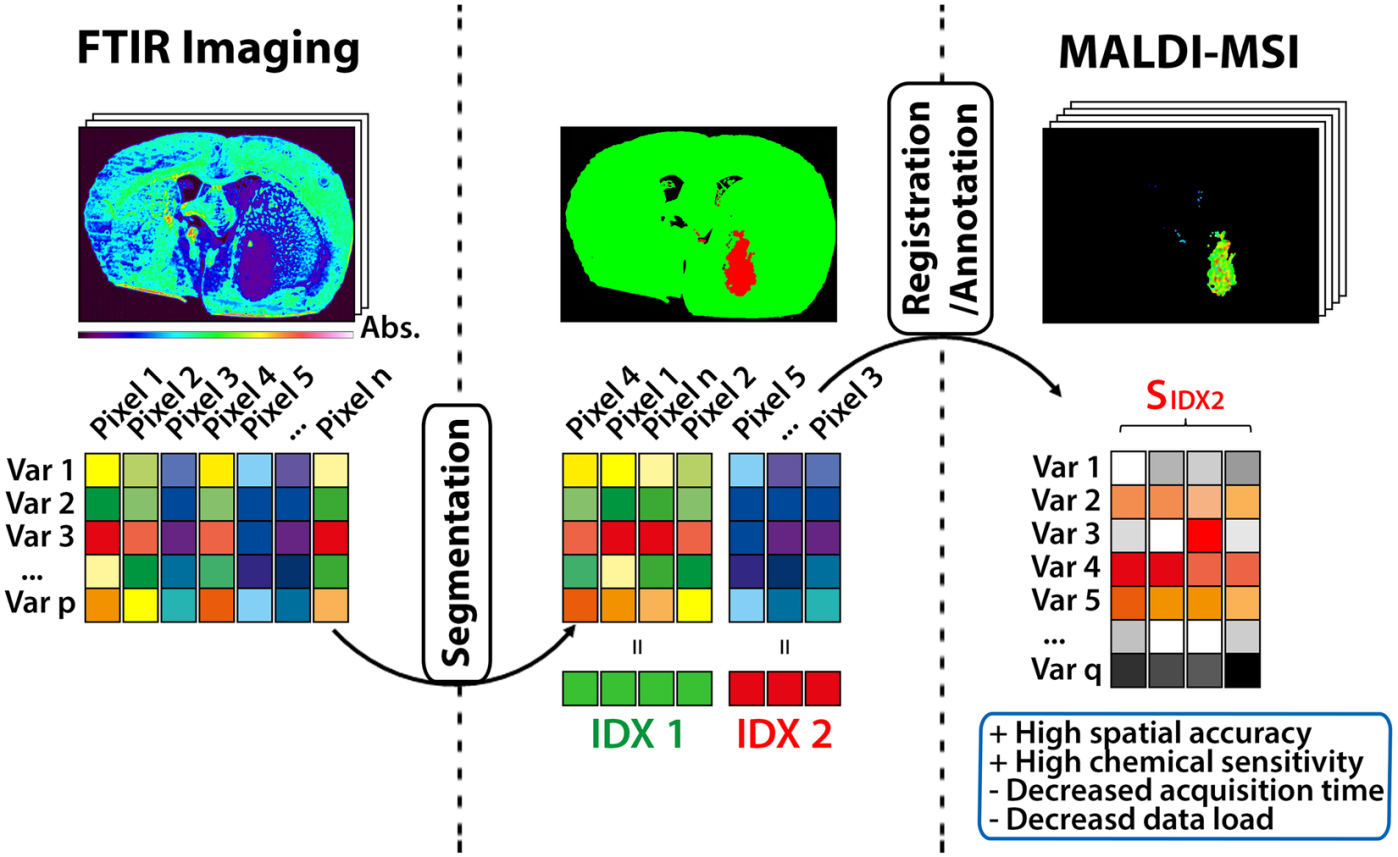
Concept of multimodal FTIR-guided MALDI mass spectrometry imaging. In a two-modality workflow, FTIR imaging is used as the first modality to record a variable (Var) set of p vibrational bands that represent molecular distribution patterns at a pixel size limit of 6.25 × 6.25 µm. By applying k-means++ segmentation, FTIR spectra of all acquired pixels are separated into a defined number of subgroups based on spectral similarities. The membership of each FTIR pixel to one of the defined groups is expressed by assigning an exclusive index number (IDX). The spatial properties of a segment belonging to a given IDX (S_IDX_) are automatically registered to the second modality, MALDI-MSI, allowing for a targeted acquisition of a greater number of q mass variables for the region of interest predefined by the FTIR subgroup. FTIR and MS imaging measure different properties of the examined tissue specimen, thus complementing contours of spatial accuracy that exceed MALDI-MSI capabilities with chemically specific mass information while reducing data load and acquisition time.

In the two-step workflow (Fig. 1), FTIR images of 8 µm tissue cryosections are acquired prior to MSI-data acquisition, processed and virtually micro-dissected via k-means++ clustering^36^, thus partitioning the tissue into multiple areas of similar molecular composition. This way, it becomes possible to localize distinct, divergent cell populations defined by molecular features, e.g. solid tumors or functionally important tissue morphologies like the hippocampus. By applying intensity-based registration, the spatial contours of each computed segment are automatically linked to the microscopic image used for subsequent MS acquisition, thus serving as a template for targeted acquisition or extraction of chemically specific mass information. Therefore, FTIR-guided MALDI-MSI enables both automated and unbiased comparison of MS signatures belonging to different IR-segments and MS image acquisition limited to a single predefined segment.

### Automated tumor-localization in MS ion images by FTIR

The localization of tumor margins is a critical task in histopathology and, hence, serves as a relevant test case for the proposed multimodal workflow. Initially we analyzed rather homogenous lesions: We inoculated brains of four female 6–12 weeks old CD1 nu/nu mice with 1 × 10^5^ human U87-MG glioblastoma cells and harvested the tumor-bearing organs 5–6 weeks after inoculation. Subsequently, we prepared adjacent coronal brain cryosections for FTIR and MALDI-MS image acquisition. For later comparison, the section used for FTIR measurement was stained with hematoxylin and eosin (H&E), and a microscopy image was taken (Suppl. Figure 1).

**Figure 2:**
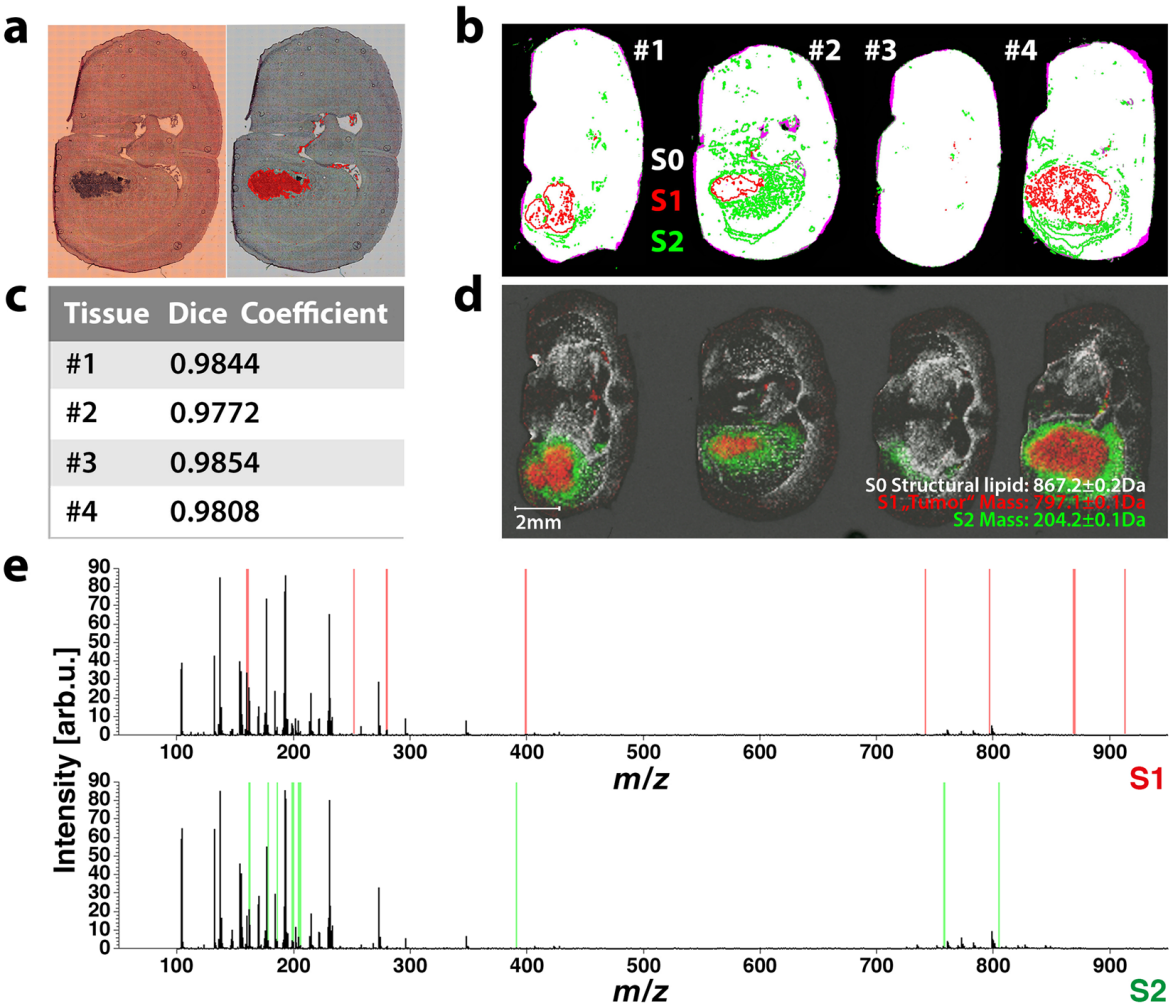
FTIR-based identification of tumor lesions in xenografted CD1 nu/nu mice. Brains of four CD1 nu/nu mice (#1 to #4) were inoculated with 1 × 10^5^ human U87-MG glioblastoma cells, and organs were harvested 5–6 weeks later. Subsequently, we prepared adjacent 8 µm coronal brain sections for FTIR and MALDI-MS image acquisition. FTIR data acquired from cryosections of all four mouse brain samples were simultaneously disaggregated by k-means++ segmentation (k = 10) to identify intra- and inter-specimen relationships. (**a**) By comparing the depicted spatial contours belonging to a given index in a single brain section (left panel) to its corresponding H&E stain on gold (right panel), it becomes possible to identify tumor-associated segments in all four specimen, because they share the same index. (**b**) Binary images derived from FTIR data and microscopic images of subsequent sections used for MS acquisition are automatically registered. The resulting fusion images (white = matching areas, magenta = non-matching areas) are overlaid by contours of two segments (S1, red & S2, green) present in all observed specimen. (**c**) The quality of registration is further evaluated using the dice similarity coefficient, which demonstrates an overlap of >97% between all transformed binary FTIR images and the binary images used for MSI measurement (**d**) Extracting only mass spectra that lie within the transformed spatial contours of S1 and S2, enables identification of m/z-values with matching distribution patterns (m/z 797.1 tumor-specific signal in S1, m/z 204.2 signal-specific for the expanded tumor margin S2) by (**e**) means of feature selection using the area between the empirical receiver operating characteristic (ROC) curve and the random classifier slope. For both, S1 and S2, ten conspicuous features (highlighted in red for S1 and green for S2) were calculated.

The FTIR images (i.e. data cubes mapping signal intensity onto [x, y, wave number] coordinates) of all four mouse brain sections were simultaneously portioned into 10 segments by virtue of a single k-means++ cluster analysis. By linking spatial properties that represent inter-and intra-section dependencies of all observed specimen with a single H&E image (Fig. 2a, right panel), it became possible to identify the segment corresponding to tumor (Fig. 2a, left panel) and thus locate tumor areas in all obtained images, as they share the same allocation. By means of intensity-based registration, the spatial properties derived from FTIR images were automatically registered to MALDI-MSI datasets. To facilitate that registration, the optical image used for MS image acquisition and all FTIR images were converted to binary images showing the presence (white) and absence (black) of tissue. After transformation, we marked the previously identified (FTIR-) segment that matched the tumor pattern (S1) and an additional segment right next to the tumor (S2), which was not noticeable in the H&E image (Fig. 2b). We speculate that this segment, an expansion of the tumor margin ‘invisible’ in H&E staining, may represent edema and/or an infiltration zone characteristic of glioma. The registered annotations were verified by calculating the Dice similarity coefficient (DSC)^37^, which suggested an overlap of at least 97% between the registered FTIR and fixed optical MS images (Fig. 2c). Translating the registered segments from FTIR into MSI coordinate space allows for the identification of chemical signals specific to the identified segments (Fig. 2d). For this, we applied feature extraction using the area between the empirical receiver operating characteristic (ROC) curve and the random classifier slope as a criterion to obtain 10 m/z values per segment that are suitable for distinguishing between outside-segment and within-segment mass spectra (Fig. 2e). For instance, we identified *m/z* 797.1 as a tumor-specific signal in S1, *m/z* 204.2 as a signal-specific for the extended tumor margin S2 and *m/z* 867.2 as an ion that was symmetrically distributed across the brain. For all identified peaks, a match between the spatial properties derived from FTIR imaging and the identified peak distribution patterns in complex MSI datasets was observed (Suppl. Figure 2). We concluded that independent detection of S2-specific features by multiple sensors verifies the authenticity of a tumor-associated area that was not observable by H&E staining and adds substantial value to exclusive MSI segmentation analyses.

**Figure 3:**
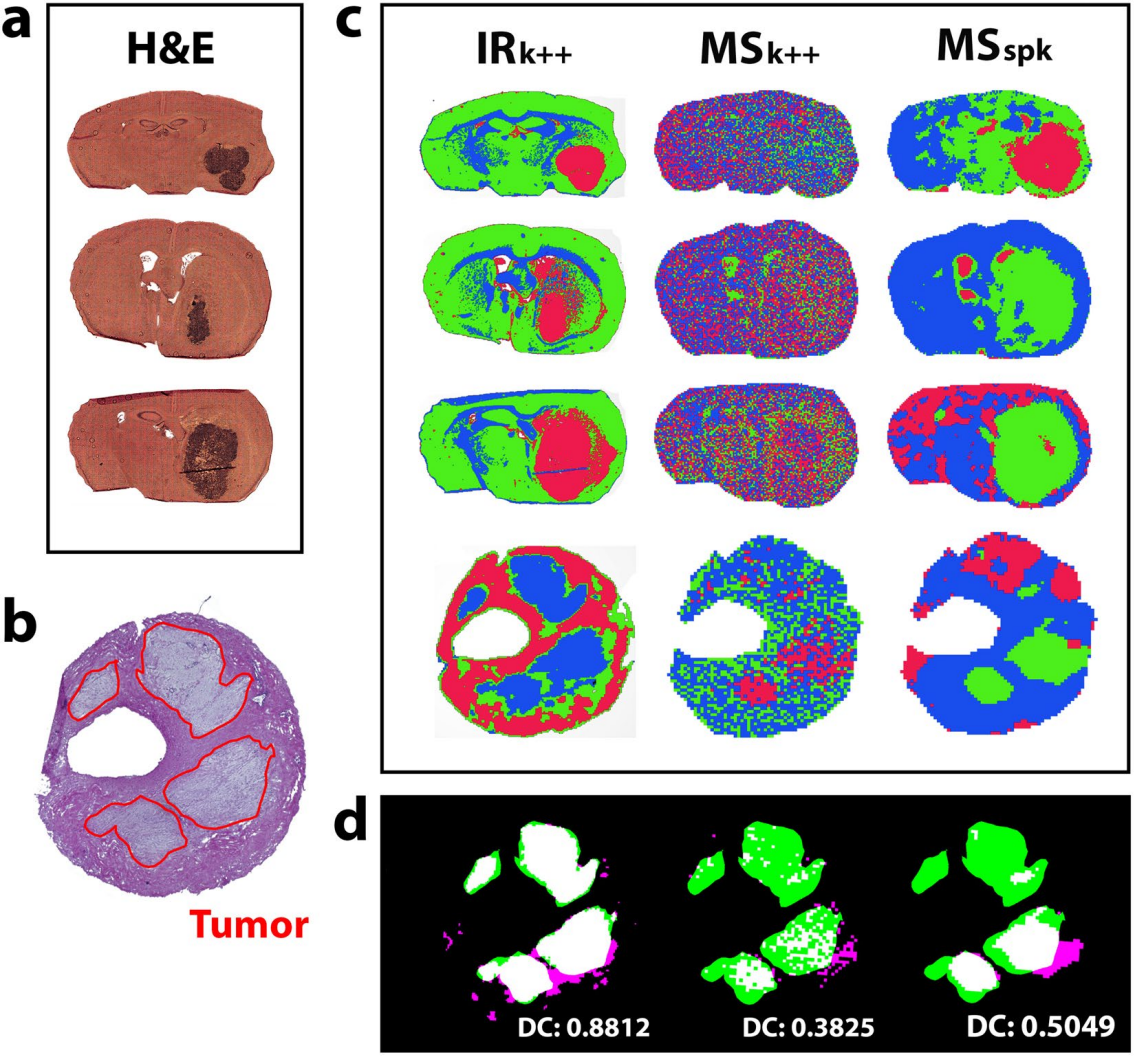
Accuracy of tumor contour assignment derived from FTIR- and MS image segmentation. **(a)** H&E stained cryosections of CD1 *nu/nu* mouse brains engrafted with U87-MG glioblastoma cells on gold-coated slides and **(b)** H&E stains of human patient-derived GIST tissue were used to evaluate **(c)** IR and MS imaging based segmentation results. The images recorded from adjacent cryosections were *in silico* dissected into three areas of greatest possible data homogeneity, expressed by a color code (red, green, blue). The resulting segmentation images derived from FTIR images using k-means++ clustering (IR_k++_) and MS images using k-means++ clustering (MS_k++_) as well as spatially aware clustering (MS_spk_) are compared. **(d)** The spatial contours of each derived segment were automatically registered to a pathologist’s tumor annotation in human GIST tissue (red circle in b) to determine each modalities’ capability of revealing the observed tumor structure. Calculation of the DSC demonstrates a more precise (88.1% overlap between the binary tumor contours and segmentation result) disaggregation of the examined tissues when based on FTIR data compared to results based on MSI data (38.3% for MS_k++_ and 50.5% for MS_spk_).

To investigate advantages of the proposed workflow further, we sought to determine the difference between FTIR segmentation for MSI-guidance as opposed to direct segmentation of MS images. We therefore expanded our initial mouse study to primary human tumors, i.e. resected tissues containing gastrointestinal stroma tumors (GIST), and histopathological stains of CD1 nu/nu mouse brain (Fig. 3a) and annotated human GIST (Fig. 3b) sections were used to evaluate segmentation results of FTIR and MS images recorded on subsequent cryosections (Fig. 3c). Recorded FTIR and MS images were grouped into three segments by k-means++ (k = 3) clustering.

Additionally, MS images were clustered using state-of-the-art spatially aware clustering methods^38^. Segmentation results for FTIR images matched tumor boundaries identified in H&E histopathology of adjacent GIST sections with higher accuracy (DSC = 0.88) when compared to segmentation results of MS images (Fig. 3d). The apparent advantage of FTIR-based versus MSI-based segmentation might reflect known challenges in MALDI-MSI such as ion suppression, diffusion of analytes, inhomogeneous matrix etc., all of which may affect the segmentation process. Moreover, simultaneous segmentation of multiple MS images is computationally challenging when compared to FTIR segmentation and only feasible after extensive data pre-processing.

### FTIR-based segmentation for exploration of tissue morphology in MSI

**Figure 4:**
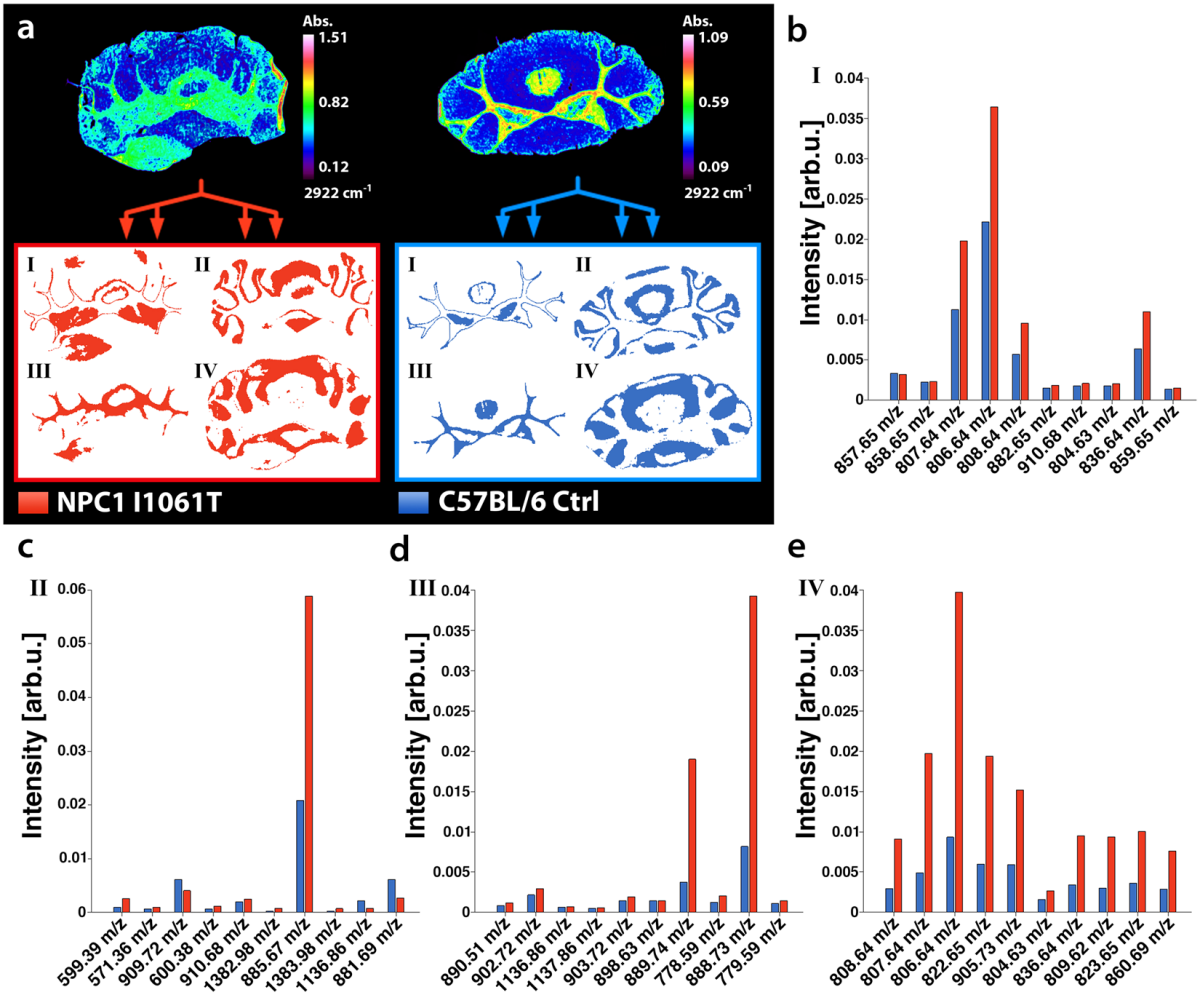
Comparison of MS lipid/metabolite signatures derived from FTIR-segmented NPC1 and Control mouse brains. **(a)** FTIR images (exemplary presentation of the absorption at 2922 cm^−1^) were recorded for coronal cerebellar sections of NPC1 I1061T knock-in and C57BL/6 control (Ctrl) mice and subsequently divided into four segments (I-IV) of maximum data similarity by means of k-means++ clustering. Comparison to the Allen Brain Reference Atlas^39^ suggests the morphological identity of three FTIR-derived segments, namely granule cell layer (II), fiber tracts (III) and molecular layer (IV). The spatial contours reflecting distinct cell morphologies were automatically transferred to MS images, in order to enable comparison between the MS lipid/metabolite profiles of NPC1 and Ctrl mice, guided to the obtained infrared segments. (**b–e**) Ten distinct *m/z* features allowing discrimination between healthy and diseased mice were identified using t-test based feature extraction revealing segment and disease-selective changes.

Comparison of FTIR images to H&E stains enable the annotation of pathologically important tissue areas, such as tumor margins. As an additional application for the proposed workflow, segment-wise comparison of tissues allows for spatially focused biomarker exploration in cases, for which no *a priori* information is available or which are inaccessible to histopathological stains. As a suitable example, we prepared coronal cryosections of cerebelli of NPC1 I1061T knock-in mice, an animal model of Niemann-Pick type C disease, featuring a spatially restricted neuronal lipid disorder and C57BL/6 control mice. FTIR images were registered and portioned into five segments, in order to in silico dissect the corresponding MSI dataset (Fig. 4a). In comparison to the Allen Brain Reference Atlas (Fig. 4b), which summarizes publicly available data about the brain’s structural makeup^39^, we could link the identified segments to their morphological counterparts. Hence, we demonstrate the dissection of FTIR images into cerebellar granular layer, molecular layer, cerebellar fiber tracts and an unspecified microstructure. The fifth calculated segment represents each tissue’s contours and was discarded for analysis. After registration (DSC = 0.98), the biomolecular signatures provided by MALDI-MSI were extracted for each individual segment to allow for a segment-wise and therefore cerebellar substructure-wise comparison of NPC1 I1061T knock-in and control mice: We observed segment-specific increases by t-test based feature extraction of the ten most discriminating mass-to-charge values. As feature extraction independently rates the discriminatory power of each mass feature, some of the retrieved masses were found to refer to isotope clusters of singly charged molecule species. This had to be expected, as the isotope peaks of a differing molecule should vary accordingly. For this reason, the provided number of potential bio signatures by our automated screening approach was less than ten. Notably, the previously described^35^ disease-related accumulation of ganglioside GM2 (d18:1/18:0) could be linked to the molecular brain layer segment. In conclusion, this investigation suggests that, without the need for H&E staining, FTIR image-based tissue segmentation and segment-wise screening for distinguishing *m/z* features within MS data can be fully automated.

### Same-slice automated high-resolution MALDI-FTICR-MSI data acquisition targeted to tissue regions of interest predefined by FTIR microscopy

So far, we have demonstrated the segmentation of FTIR images into histologically comparable regions, thus allowing for an improved exploration of subsequent MSI data. Although the presented workflow is advantageous for tissue exploration purposes and automated feature retrieval, it introduces additional data acquisition time and data load. This approach is especially unfavorable for rather slow high-resolution MALDI-MSI using an FTICR detector for which these factors represent a significant bottleneck.

**Figure 5:**
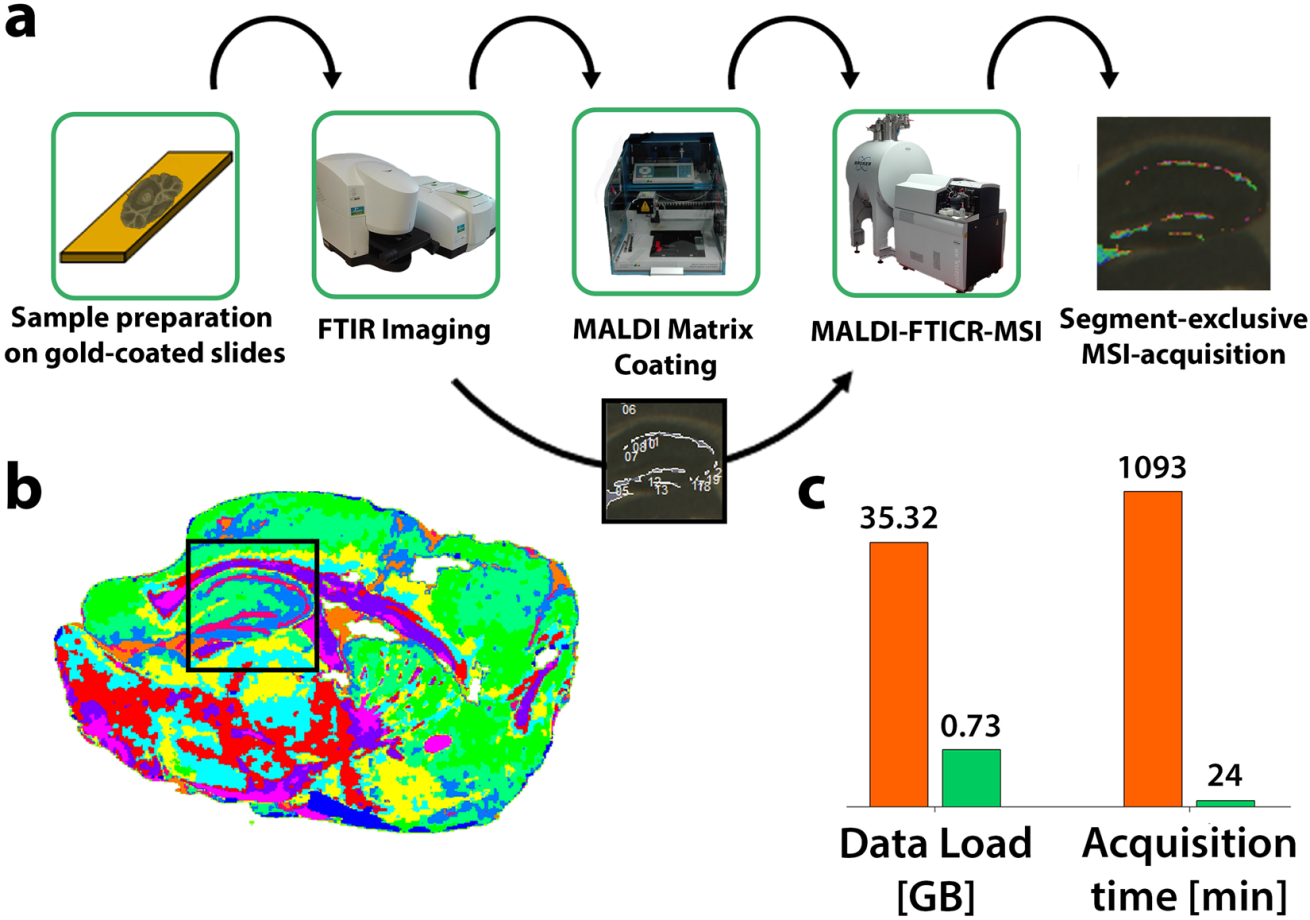
FTIR-guided, spatially restricted data acquisition by high-resolution, MALDI-FTICR-MSI. (**a**) Multimodal workflow for the consecutive FTIR and MALDI-FTICR-MSI acquisition of a single slide. The samples to be tested are mounted on gold-coated microscope slides for their reflective qualities needed for FTIR and conductive properties needed for MS imaging. Slides are briefly dried and passed to the FTIR microscope. (**b**) Based on the resulting image, segments are calculated based on k-means++ cluster analysis and used for later MSI-guidance. Meanwhile, an optical image is taken and matrix solution is sprayed on the specimen. The spatial features of a particular FTIR derived segment are registered to the optical image used for subsequent MALDI-FTICR-MSI, thus enabling the targeted segment-exclusive recording of mass features. (**c**) Predefined MALDI-FTICR-MSI acquisition of the granular cell layer found in the hippocampus of a sagittal C57BL/6 mouse brain section using a 512k data transient significantly decreases data load (97.9%) and acquisition time (97.8%).

We therefore modified our multimodal measurement pipeline (Fig. 5a) to allow for automated multimodal acquisition on the same tissue section. Most importantly, transfer of FTIR-derived spatial information to the FTICR mass spectrometer before MSI acquisition enables the exclusive measurement of pre-defined single segments in subsequent MALDI-FTICR-MSI. To examine the capabilities of this refined workflow, we prepared sagittal brain sections from C57BL/6 wildtype mice for FTIR- followed by MALDI-FTICR-MSI measurement. Utilizing FTIR image-based segmentation, MALDI-FTICR-MSI measurement was guided to the dentate gyrus of the hippocampus using a common 512kB data point transient (Fig. 5b). The resulting image was acquired in 25 min, which indicates a reduction of 97.8% compared to an acquisition time of 18.3 h for the whole tissue section (Fig. 5c). Accordingly, the data volume of the whole tissue section (35.3 GB) was reduced to 0.7 GB. Not surprisingly, time saving and data load decrease are determined by the size of the respective segment used for acquisition. The technique enables targeted high-resolution MALDI-FTICR-MSI in cases where the acquisition of whole tissue sections is not desired or feasible.

## Discussion

The workflow presented here for FTIR-guided high-resolution MSI offers interpretation and data acquisition focused on defined tissue sub-compartments independent of histopathological staining. This represents a substantial advantage when compared to previous image fusion approaches^12,14,40^ that rely on H&E images of adjacent sections for interpretation. Data acquisition on the same tissue section represents a significant upgrade by further improving registration efficiency and minimizing deficiencies caused by sectioning artifacts, such as tears and folds. While the registration of adjacent sections for the most part depends on the operating scientist and can therefore vary^14^, registration of images taken from a single specimen is more reliable. Utilizing spatial features derived from FTIR imaging, we provide three innovative approaches for multimodal data acquisition and interpretation. First, we demonstrate the precise identification of tumor-associated contours in a single segmentation of multiple cancer tissues, thus enabling tumor annotation in all recorded tissue sections on the basis of a single H&E stain. Whereas the capability of visualizing sparse tumor populations by FTIR imaging still presents a challenge, automated segmentation of lesions was readily done. Interestingly, we observed molecular differences compared to normal tissue in an expansion of the tumor margin (‘S2 segment’) detected by both FTIR and MALDI-MSI. This segment was ‘H&E-invisible’ suggesting that it may represent a part of the tissue that was morphologically non-cancerous, but molecularly substantially altered.

Although FTIR imaging provides less chemically specific data when compared to MALDI-MSI, FTIR-based segmentation holds significant advantages as it is unaffected by ion suppression, analyte diffusion or other effects that make MSI-based segmentation challenging. The presented concept for multimodal imaging can be easily transferred to any other non-destructive optical upstream modality (i.e. Raman imaging), thus providing guidance in cases where FTIR imaging is incapable of identifying the region of interest. Spatially guided imaging is applicable to MSI data obtained using any ionization method and mass spectrometer (i.e. DESI-MSI, SIMS-MSI). The only requirement is the alignment of both imaging planes onto the same coordinate system. In cases for which information about the spatial occurrence of disease-related effects is lacking, we presented FTIR-based segmentation for automated tissue morphology exploration in MSI. Spatial decomposition occurs solely at data level and holds decisive advantages when compared to disruptive procedural steps such as laser-capture microdissection. Without any *a priori* information, specific mass shifts could be detected and linked to cerebellar subsections in an unbiased fashion. Biomedical MSI is typically not feasible for intraoperative applications and therefore carried out in analytical laboratories for which tissue annotation is a major bottleneck. The automatic alignment and dissection of MS data into subsections not only facilitates comparative studies of structured organs (e.g. brain, kidney), but also enables spatially aware biomarker identification. Serving as an example, we presented segment-wise MS data comparison in mouse brain, arguably one of the most-used models to investigate human neurological diseases^41^. Segment-specific MALDI-MSI not only enables the exclusive analysis of disease-related areas, but also enables the exclusive processing of similar cell populations, thus reducing data variance when compared to whole-organ images. Simultaneous segmentation of multiple sections enables the comparative analysis of sub-compartments of similar morphological makeup without the need of histopathological examination. Lastly, we demonstrated FTIR-denoted, segment-exclusive MALDI-FTICR-MSI as a way to overcome the compromise between pixel size and spectral resolution needed for high-throughput measurements. This application aims for very specific analyses of tissue subsections as time and data savings are achieved by discarding the pixels of all remaining sections before acquisition.

The capability to identify and exclusively acquire small-sized tissue compartments is ultimately denoted by the guiding optical modality and clustering algorithm. As the FTIR image recording first introduces additional time and data load, savings are only achieved if they fall below the costs to obtain all image pixels in subsequent MSI, thus limiting this particular approach to very slow detectors. Depending on section size and resolution, recording of FTIR images took on average one hour which is why no major lipid degradation was expected. It may, however, occur during longer processing times. Additionally, the current workflow implementation does not account for the occurrence of isotopic peaks within the MSI data which would be preferred to enhance marker signature retrieval. This issue could be resolved by adding computational de-isotoping measures to the presented workflow.

While segmentation and registration are automated, the value k needed for k-means++ clustering represents a remaining source for user-bias. The current work has focused on k-means++ clustering for its potential to rapidly dissect complex data. However, alternate segmentation tools like agglomerative hierarchical clustering^24^ could enable a more detailed and independent coverage of tissue areas at the cost of central processing unit (CPU) load.

Although the retrieved segments correlate to anatomical tissue structures in brain atlas data, cluster-based tissue dissection currently does not provide direct labeling of the retrieved segments. However, exploration of tissue morphologies can still be performed. Simultaneous clustering thereby ensures comparability by identifying spectral similarities across multiple sections.

In conclusion, this multimodal workflow combines the advantages of FTIR and MALDI-MSI technologies to provide a deeper biological understanding of tissue structure. It also allows the exclusive acquisition of regions of interest to increase throughput in cases where the acquisition of whole tissue sections at high resolution is impractical or even infeasible.

## Materials and Methods

### Animal studies and human tissue specimen

Animal studies on engrafted CD1 nu/nu conducted at the German Cancer Research Center (DKFZ, Heidelberg) were supervised by institutional animal protection officials in accordance with the National Institute of Health guidelines Guide for the Care and Use of Laboratory Animals. The animal experiments were approved by governmental authorities (Regierungspräsidium Karlsruhe, Germany, approval number: G187/10). Four female 6–12 weeks old CD1 nu/nu mice were inoculated with 1 × 10^5^ human U87 glioblastoma cells. All mice were sacrificed when the first mouse became symptomatic (5–6 weeks after inoculation), and brain tissue samples were snap-frozen^33^. Lipid signatures were analyzed in cerebellar sections of Niemann-Pick C1 I1061T knock-in mice^35^ provided by the Ory laboratory at Washington University, St. Louis, USA. Mice were kept in a controlled animal facility and given standard chow and water ad libitum. Weaning occurred between 3 and 4 weeks. Experimental procedures were approved by the Washington University Animal Studies Committees and were conducted in accordance with the USDA Animal Welfare Act and the Public Health Service Policy for the Humane Care and Use of Laboratory Animals.

Mass signatures were also analyzed in human GIST samples provided by the University Medical Centre Mannheim. Informed consent was obtained in all cases from patients whose tissues were used in this study. Experiments were performed in accordance with applicable laws and regulations, good clinical practices and approved by an independent ethics committee (“Medizinische Ethik-Kommission II” of Heidelberg University; No. 2012-293N-MA).

### Tissue preparation and evaluation

For all experiments, 2–3 adjacent sections of 8 μm thickness were cut from the cryopreserved tissue specimen by using a CM 1950 cryostat (Leica Biosystems, Nussloch, Germany). Tissue slides designated for multimodal measurements on single sections were placed on gold-coated slides (Science Services, Munich, Germany). Sections designated for exclusive MALDI-MSI were mounted on indium-tin-oxide (ITO)-coated glass slides (Bruker Daltonics, Bremen, Germany). All slides were dried in a desiccator overnight at RT and stored at −80 °C. Reference sections for pathological annotation at the Pathology Institute (UMM) were mounted on Starfrost adhesive microscope slides (R. Langenbrinck GmbH, Emmendingen, Germany), stained using hematoxylin & eosin (H&E) and scanned using an Aperio CS2 bright field scanner (Leica Biosystems, Nussloch, Germany).

### FTIR Imaging

Mid-infrared images were recorded using a Spotlight 400 FT-IR Imaging System (PerkinElmer, Waltham, USA). Measurement and processing parameters were specified by using the Spectrum^TM^ Image software (PerkinElmer). For annotation and virtual dissection experiments, 2 co-added scans per 25 × 25 μm pixel were recorded in the range of 4000–650 cm^−1^ with a spectral resolution of 8 cm^−1^. Images recorded for exclusive FTIR-guided MALDI-FTICR MSI featured 2 co-added scans per 6.25 × 6.25 μm pixel in the range of 3200–750 cm^−1^ with a spectral resolution of 12 cm^−1^. All images were acquired in reflection mode with a mirror velocity of 2.2 cm/s. Fourier transform integration was done by selecting the Norton-Beer function for apodization.

### MS Imaging

MALDI matrix was applied on all tissue sections using the SunCollect automatic sprayer (SunChrom, Friedrichsdorf, Germany) as described previously^42^. For positive-ion mode measurements, five layers (ascending flow rates of 10, 15, 3 × 20 μL/min) of DHB solution (60 mg/mL 2,5-dihydroxybenzoic acid in 50% acetonitrile and 0.5% trifluoroacetic acid) were sprayed on the tissue. Tissues designated for negative-ion mode measurements were sprayed with nine layers (at 1 × 10 μL/min, 1 × 15 μL/min, 1 × 20 μL/min, 6 × 25 μL/min) of PhCCAA solution (5 mg/mL 4-Phenyl-α-cyanocinnamic acid amide in 90% acetone). MALDI-MSI experiments were performed on an ultrafleXtreme mass spectrometer (Bruker Daltonics). Data acquisition parameters were specified in flexControl (Bruker Daltonics), using 500 laser shots per position with a laser raster width of 75 μm for the acquisition of CD1 nu/nu mice brain sections and 50 μm for the acquisition of NPC1 I1061T knock-in mice sections. For calibration, the Bruker peptide calibration standard (Bruker Daltonics) was used. High-resolution data were acquired on a SolariX 7 T XR MALDI-FTICR mass spectrometer (Bruker Daltonics). Spectra were recorded in negative-ion mode within the m/z range 150–4000 using absorption mode and a 512kB data point transient. Data points were acquired using a pixel size of 20 μm and 40 laser shots per pixel. Data acquisition parameters were specified in ftmsControl (Bruker Daltonics). Measurement areas and ROIs for both MALDI-MSI and MALDI-FTICR-MSI analyses were visualized in flexImaging 4.1 (Bruker Daltonics).

### Pre-processing pipeline

A workflow for optimized FTIR image pre-processing and information recovery was established in MATLAB (The MathWorks, Natick, USA). FTIR spectra were baseline-corrected using asymmetric least squares smoothing presented by Eilers and Boelens^43^. Next, corrected spectra were subjected to first derivative calculation and standard normal variate (SNV) normalization. Spectral windows within the ranges 3100–2900 cm^−1^ and 1800–950 cm^−1^ were selected. After spectral pre-processing, the hyperspectral imaging data cube was subjected to spatial pre-processing. Thereby, edge-preserving denoising (EPD)^44^ was performed by courtesy of a MATLAB function provided by Dr. Markus Grasmair^45^ and pixels acquired on tissue-free slide surfaces were removed. For MS data, peak picking based on finding local maxima was performed prior to feature extraction using the ‘rankfeature’ function in MATLAB based on the Area between the empirical receiver operating characteristic (ROC) curve and the random classifier slope. The pre-processing of the MSI data was carried out in R using the ‘MALDIquant’^46^ package with TIC normalization, Top Hat baseline correction and peak picking with S/N > 3 using the Friedman’s Supersmoother^47^ method. The spatially aware segmentation of the MSI data was carried out as per the implementation available in the ‘Cardinal’ package^48^ using Gaussian weighting with pixel neighborhood radius of 3 pixels.

### Multimodal annotation and registration tool

Segmentation of the hyperspectral FTIR data cube was achieved by applying the k-means++^36^ MATLAB function of the Statistics and Machine Learning toolbox. Subsequently, the recorded FTIR images were aligned to their corresponding tissue objects, visible in the optical image used for MSI acquisition, by means of affine registration (translation, rotation, scaling). In a preliminary step, the tissue shapes within the FTIR and optical image were converted into binary files by k = 2 cluster analysis. We performed intensity-based image registration in MATLAB to minimize the mean squared error between the moving FTIR-segment and the fixed binary image derived from the optical scan. For evaluation of registration accuracy, the DSC was calculated between the warped FTIR-derived binary image X and the fixed binary image Y originating from the optical image used for MSI acquisition.

### Data availability statement

FTICR datasets generated during and/or analyzed during the current study are available in the EU-METASPACE repository [http://metaspace2020.eu/]. Other datasets generated during and/or analyzed during the current study are not publicly available but are available from the corresponding author on reasonable request.

## Electronic supplementary material

Supplementary Information
