# Author Correction: Fourier Transform Infrared Microscopy Enables Guidance of Automated Mass Spectrometry Imaging to Predefined Tissue Morphologies

**DOI:** 10.1038/s41598-018-24807-z

**Published:** 2018-04-18

**Authors:** Jan-Hinrich Rabe, Denis A. Sammour, Sandra Schulz, Bogdan Munteanu, Martina Ott, Katharina Ochs, Peter Hohenberger, Alexander Marx, Michael Platten, Christiane A. Opitz, Daniel S. Ory, Carsten Hopf

**Affiliations:** 1 Center for Applied Research in Applied Biomedical Mass Spectrometry (ABIMAS), Mannheim University of Applied Sciences, Paul-Wittsack Str. 10, 68163 Mannheim, Germany; 2 Institute of Medical Technology, Heidelberg University and Mannheim University of Applied Sciences, Paul-Wittsack Str. 10, 68163 Mannheim, Germany; 3 German Cancer Consortium (DKTK) CCU Neuroimmunology and Brain Tumor Immunology, German Cancer Research Center (DKFZ), Heidelberg, Germany; 4 University Medical Center Mannheim of Heidelberg University, Mannheim, Germany; 5 Brain Cancer Metabolism Group, German Cancer Research Center (DKFZ), Heidelberg, Germany; 6 Department of Neurology and National Center of Tumor Diseases, University Hospital Heidelberg, Heidelberg, Germany; 7 Diabetic Cardiovascular Disease Center and Department of Medicine, Washington University School of Medicine, St. Louis, Missouri 63110 USA

Correction to: *Scientific Reports* 10.1038/s41598-017-18477-6, published online 10 January 2018

In the original version of this Article, Katharina Ochs was incorrectly affiliated with:

German Cancer Consortium (DKTK) CCU Neuroimmunology and Brain Tumor Immunology, German Cancer Research Center (DKFZ), Heidelberg, Germany.

and

Brain Cancer Metabolism Group, German Cancer Research Center (DKFZ), Heidelberg, Germany.

The correct affiliations are below.

German Cancer Consortium (DKTK) CCU Neuroimmunology and Brain Tumor Immunology, German Cancer Research Center (DKFZ), Heidelberg, Germany.

and

Department of Neurology and National Center of Tumor Diseases, University Hospital Heidelberg, Heidelberg, Germany.

