# Supplementary material for: Fourier Transform Infrared Microscopy Enables Guidance of Automated Mass Spectrometry Imaging to Predefined Tissue Morphologies
Source: Sci Rep. 2018 Jan 10;8:313. doi: 10.1038/s41598-017-18477-6 (PMC5762902; doi:10.1038/s41598-017-18477-6)
Supplement: Supplementary file 1 — Supplementary Information [file 41598_2017_18477_MOESM1_ESM.pdf]

Supporting Information (SI) for

Fourier Transform Infrared Microscopy Enables Guidance of Automated Mass Spectrometry Imaging to Predefined Tissue Morphologies

Jan-Hinrich Rabe, Denis A. Sammour, Sandra Schulz, Bogdan Munteanu, Martina Ott, Katharina Ochs, Peter Hohenberger, Alexander Marx, Michael Platten, Christiane A. Opitz, Daniel S. Ory and Carsten Hopf

Supplemental Figures and Figure Legends

**Supplemental Figure 1**	Spatial correlation of H&E stained Images and FTIR-derived segments of coronal mouse brain sections
**Supplemental Figure 2**	Distribution patterns of mass peaks identified as most suitable for FTIR-derived segment representation

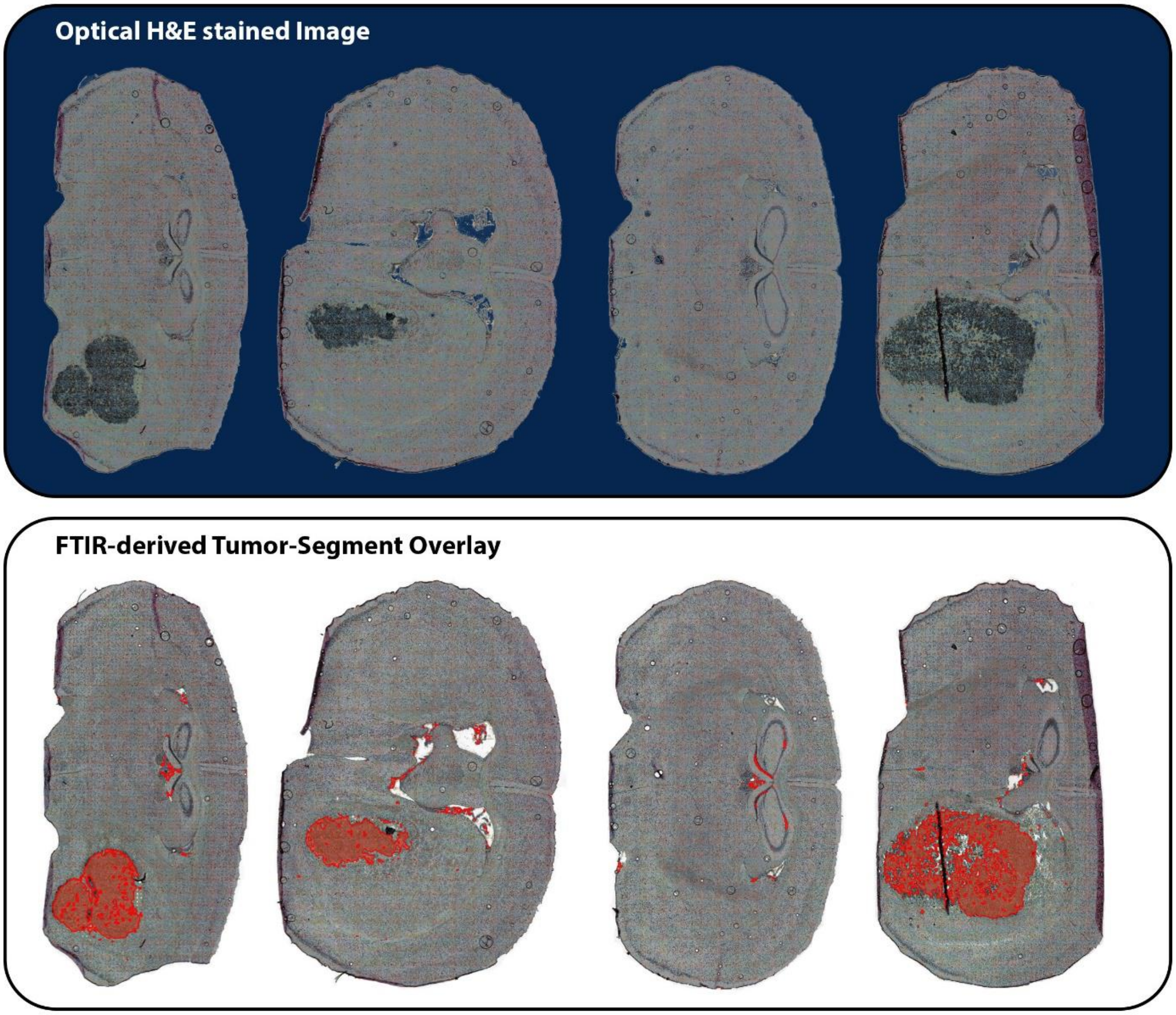

Supplemental Figure 1. Spatial correlation of histopathological H&E stained images and FTIR-derived tumor contours registered to the optical mouse brain section image. Coronal mouse brain cryosections of CD1 nu/nu mice engrafted with U87-MG glioblastoma cells were prepared for subsequent FTIR imaging (upper panel) and H&E staining (lower panel). Using k-means++ clustering (k=10), FTIR spectra of all four mouse brain sections were simultaneously dissected into subgroups of spectral similarity. The spatial properties of the tumor-representing subgroup were automatically registered to the corresponding optical image (Mean Dice similarity coefficient: 0.994).

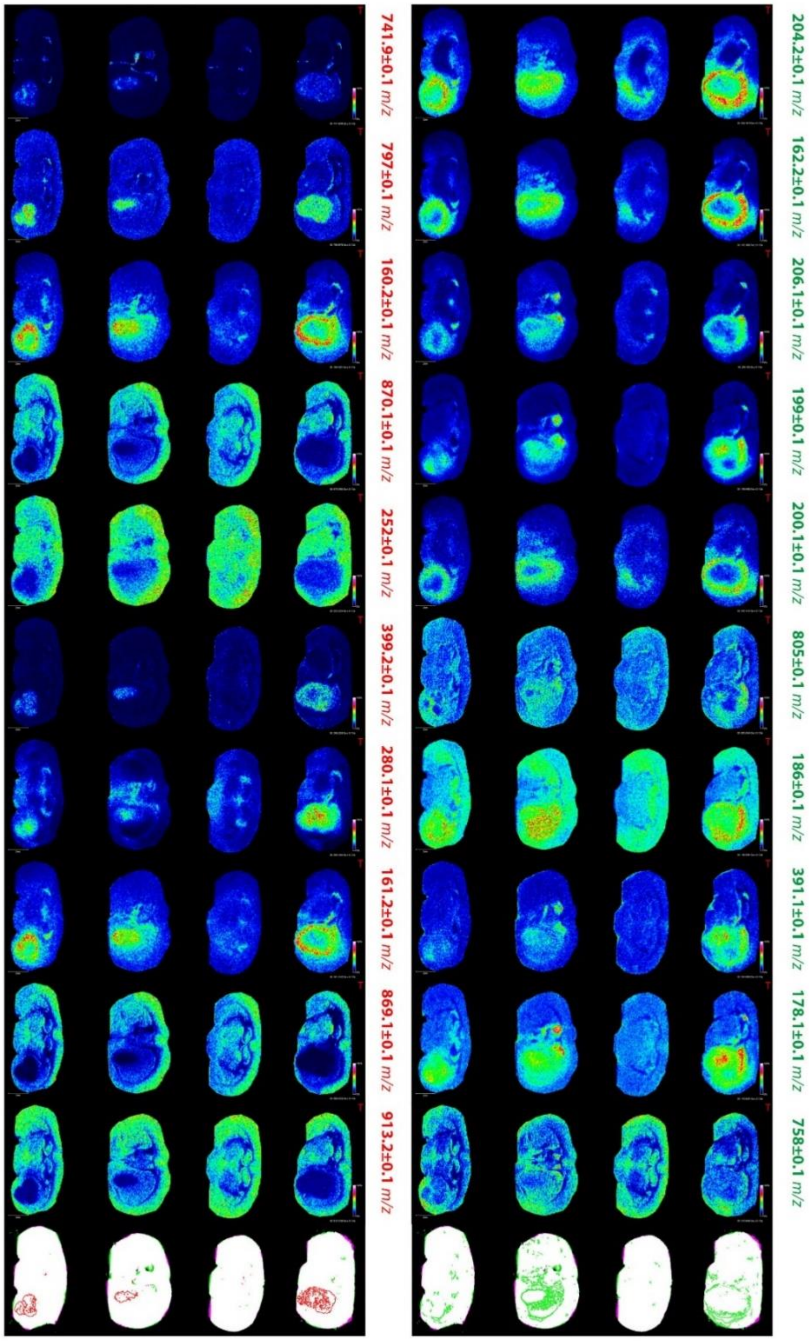

Supplementary Figure 2. Distribution patterns of mass peaks identified as most suitable for FTIR-derived segment representation. Subsequent to MSI, FTIR-Images of CD1 *nu/nu* mice brain sections engrafted with U87-MG glioblastoma cells were acquired and clustered for MS-independent identification of tumor-associated spatial distribution patterns. Out of the identified segments, two were considered to be related to tumor and their spatial distribution pattern was marked in all four recorded specimen (bottom row, red: tumor-segment S1, green: “extended tumor margin”-segment S2). For each segment, the 10 most discriminant m/z values were identified within the MSI data cube by means of feature extraction using the area between the empirical ROC curve and the random classifier slope as a criterion. The distribution patterns of the identified mass values (left panel: S1, right panel: S2) support the spatial arrangement of both FTIR-derived segments.
